# Gold Kiwi-Derived Nanovesicles Mitigate Ultraviolet-Induced Photoaging and Enhance Osteogenic Differentiation in Bone Marrow Mesenchymal Stem Cells

**DOI:** 10.3390/antiox13121474

**Published:** 2024-11-29

**Authors:** Doyeon Kim, Chanho Lee, Manho Kim, Ju Hyun Park

**Affiliations:** 1 Department of Biomedical Science, Kangwon National University, Chuncheon-si 24341, Gangwon-do, Republic of Korea; rlaehdus6321@kangwon.ac.kr (D.K.); 202216496@kangwon.ac.kr (C.L.); manhokim@kangwon.ac.kr (M.K.); 2 Institute of Molecular Science and Fusion Technology, Kangwon National University, Chuncheon-si 24341, Gangwon-do, Republic of Korea

**Keywords:** gold kiwi-derived extracellular nanovesicles (GK-NVs), bone marrow mesenchymal stem cells (BM-MSCs), photoaging, oxidative stress, bone regeneration

## Abstract

Bone marrow mesenchymal stem cells (BM-MSCs) play a crucial role in bone formation through their ability to differentiate into osteoblasts. Aging, however, detrimentally affects the differentiation and proliferation capacities of BM-MSCs, consequently impairing bone regeneration. Thus, mitigating the aging effects on BM-MSCs is vital for addressing bone-related pathologies. In this study, we demonstrate that extracellular nanovesicles isolated from gold kiwi (GK-NVs) protect human BM-MSCs from ultraviolet (UV)-induced photoaging, thereby alleviating aging-related impairments in cellular functions that are crucial for bone homeostasis. Notably, GK-NVs were efficiently taken up by BM-MSCs without causing cytotoxicity. GK-NVs reduced intracellular reactive oxygen species (ROS) levels upon UV irradiation, restoring impaired proliferation and migration capabilities. Furthermore, GK-NVs corrected the skewed differentiation capacities of UV-irradiated BM-MSCs by enhancing osteoblast differentiation, as evidenced by the increased expression in osteoblast-specific genes and the calcium deposition, and by reducing adipocyte differentiation, as indicated by the decreased lipid droplet formation. These findings position GK-NVs as a promising biomaterial for the treatment of bone-related diseases such as osteoporosis.

## 1. Introduction

Bone marrow mesenchymal stem cells (BM-MSCs) represent a cornerstone in regenerative medicine due to their distinctive ability to differentiate into a variety of cell types, including osteoblasts, chondrocytes, and adipocytes [1]. Considering the complicated relationship between aging and regenerative capacity, a comprehensive exploration reveals the multifaceted impact of aging on BM-MSCs. The aging process encompasses a series of cellular and molecular deteriorations, including DNA damage, telomere attrition, epigenetic alterations, and mitochondrial dysfunction, among others. These factors contribute to cellular senescence and stem cell exhaustion, leading to a decline in the regenerative potential and proliferation of stem cells [2]. Specifically, the aging machinery in BM-MSCs involves genomic destabilization, the disruption of proteostasis, and perturbations in intracellular signaling pathways, collectively culminating in cellular aging [3]. Cellular senescence, characterized by a stable cell cycle arrest, curtails the proliferative capacity of cells due to the accumulated intercellular damage, notably oxidative stress-dependent DNA damage [4]. Genomic instability and telomere shortening emerge as the primary activators of BM-MSC aging, with the former characterized by a compromised DNA repair capability and increased susceptibility to tumorigenesis and genomic aberrations [5]. This susceptibility underscores the critical need for quality control protocols before the clinical application of BM-MSCs so to ensure genetic stability and to avoid potential adverse outcomes. In addition, the aging process imposes a significant challenge by modulating the differentiation capacity of BM-MSCs, skewing the balance from osteogenesis towards adipogenesis [6,7]. This shift assumes a critical factor in the onset of age-related osteoporosis, a condition influenced not only by hormonal changes such as estrogen decline but also by intrinsic aging processes inherent to BM-MSCs, affecting both sexes [8].

In the rapidly advancing field of biotechnology, the exploration of extracellular vesicles (EVs) has emerged at the forefront of novel therapeutic strategies. EVs, characterized by their unique lipid bilayers, facilitate the efficient transport of materials within and across cellular membranes, exhibiting remarkable stability and permeability [9]. Notably, plant-derived EVs (pEVs) are particularly promising due to their cost-effective mass production and inherent biocompatibility, attributed to the long-standing use of plants in human applications without eliciting immune responses [10]. Moreover, pEVs also exhibit efficient cellular uptake, being internalized by cells within 12 h. This rapid uptake is attributed to their lipid bilayer membranes composed of sphingolipids, phospholipids, sterols, and other lipids, similar to those found in mammalian-derived EVs [11,12,13,14]. Furthermore, these stable pEVs can be stored for extended periods at temperatures as low as −80 °C while maintaining their structural integrity in various biological environments, including in vitro models simulating the pH conditions of the stomach and small intestine [15,16,17]. Many studies have demonstrated the therapeutic potential of pEVs and their functions on various cellular activities. For instance, EVs extracted from grapefruit were shown to enhance cell proliferation and migration while reducing intracellular reactive oxygen species (ROS) production in human epidermal keratinocytes [18]. Perut et al. reported that strawberry-derived EVs were taken up by human adipose-derived stem cells while delivering both small RNA and high levels of vitamin C, and mitigated oxidative stress [19]. In addition, a study has indicated that ginger-derived EVs are capable of being internalized by macrophages, and can suppress the activation of the nucleotide-binding domain and the leucine-rich repeat-containing family, pyrin domain-containing 3 (NLRP3) inflammasome, which plays an important role in innate immune responses [20]. Our recent investigations also reveal the therapeutic potential of EVs derived from *Aloe saponaria* and balloon flower roots, which demonstrate antioxidative and anti-inflammatory activities [21,22].

In this study, we explored the potential of pEVs in modulating physiological activities and treating diseases, with a special focus on the use of gold kiwi-derived extracellular nanovesicles (GK-NVs). Gold kiwi, known for its health-beneficial properties due to its rich antioxidant content, including vitamin C, vitamin E, and polyphenols, serves as the foundation of our investigation [23,24]. This study specifically examined its role in regulating intracellular ROS levels, with a focus on aging processes through the interplay between ROS and aging mechanisms [25]. We hypothesized that GK-NVs could represent an innovative approach to mitigating ultraviolet (UV)-induced, aging-related alterations in BM-MSCs and improve their differentiation potential toward osteoblasts. By employing polyethylene glycol (PEG) precipitation methods for EV isolation and characterizing their physical properties, we investigated the effects of GK-NVs on ROS levels, cell migration, proliferation, and the differentiation trajectory of aged human BM-MSCs between osteogenesis and adipogenesis pathways. The objective of this study is to elucidate the therapeutic potential of pEVs, specifically GK-NVs, in mitigating the adverse effects of aging on the differentiation capacity of BM-MSCs, with broader implications for the management of age-related disorders in bone regeneration.

## 2. Materials and Methods

### 2.1. Isolation of GK-NVs

Gold kiwi (*Actinidia chinensis* Planch.) was purchased from a local market in South Korea. To obtain the GK-NVs, gold kiwi fruit was peeled and thoroughly washed with distilled water. The fruit was then homogenized in phosphate-buffered saline (PBS) using a blender, and the mixture was sequentially centrifuged at 1000× *g* for 10 min, 3000× *g* for 30 min, and 10,000× *g* for 1 h to eliminate large debris. The supernatant was treated with 8% PEG (Sigma-Aldrich, St. Louis, MO, USA) and incubated at 4 °C for 16 h, facilitating the precipitation of GK-NVs by centrifugation at 1500× *g* for 30 min. The pellet obtained was resuspended in PBS to isolate the GK-NVs, which were then aliquoted and stored at −80 °C until further experiments.

### 2.2. Characterization of GK-NVs

Nanoparticle tracking analysis (NTA) was conducted using a NanoSight NS300 (Malvern Panalytical, Malvern, UK) to determine the size distribution and concentration of the GK-NVs. Their purity was assessed via a BCA assay (Thermo Fisher, Waltham, MA, USA) to quantify the total protein content, including the membrane proteins, of the EVs. To further evaluate the purity, the number of particles determined by the NTA was normalized by the total protein content measured by the BCA assay. Dynamic light scattering (DLS) analysis was performed using a Zetasizer Nano ZS (Malvern Panalytical) to measure the zeta potential and polydispersity index (PDI) values of the GK-NVs. The morphology of the isolated GK-NVs was examined by transmission electron microscopy (TEM). Briefly, an appropriate amount of GK-NVs was placed on carbon-coated copper grids (Ted Pella Inc., Redding, CA, USA). The GK-NVs on the grids were stained with uranyl acetate, and excess stain was removed by washing with distilled water. The stained GK-NVs were visualized using a CM120 electron microscope (Philips, Amsterdam, The Netherlands).

### 2.3. Cell Culture

The BM-MSCs were obtained from Promocell. The cells were cultured in growth medium (MGM2, Promocell, Heidelberg, Germany) according to the manufacturer’s instructions, with only cells with a passage number of seven or fewer being used in all experiments. All cell cultures were conducted at 37 °C in a humidified atmosphere with 5% CO_2_.

### 2.4. Evaluation of Cytotoxicity and Cellular Uptake of GK-NVs

The cytotoxicity of the GK-NVs on the BM-MSCs was evaluated using the trypan blue exclusion assay. The BM-MSCs were plated at a density of 1 × 10^4^ cells/cm^2^ in 24-well plates (SPL, Pocheon, Republic of Korea) pre-coated with 0.2% gelatin and allowed to adhere for 24 h. Subsequently, the cells were treated with various concentrations of GK-NVs in serum-free DMEM/F12 medium (Thermo Fisher) for either 24 h or 48 h. After the treatment, live and dead cells were counted under a microscope (Leica, Bensheim, Germany) following the trypan blue staining. To verify the cellular uptake, the BM-MSCs were treated with GK-NVs labeled with PKH67 fluorescence dye (Sigma-Aldrich). After a 12 h of incubation in a CO_2_ incubator, and subsequent nuclei staining with the Hoechst 33342 (Thermo Fisher), the cells were observed under a fluorescence microscope (Leica).

### 2.5. Assessment of Intracellular ROS Levels

The effect of the GK-NVs on the intracellular ROS levels in the BM-MSCs was assessed using 2′,7′-dichlorodihydrofluorescein diacetate (H2DCFDA, Sigma-Aldrich). BM-MSCs cultured in 24-well plates were treated with GK-NVs in serum-free DMEM/F12 for 24 h, followed by repeated washing with PBS. The cells were then exposed to 80 mJ/cm^2^ of UVB irradiation using a Bio-Sun illuminator (Vilber Lourmat, Eberhardzell, Germany), and subsequently incubated in DMEM/F12 for an additional 24 h. To visualize the intracellular ROS, the cells were incubated with H2DCFDA for 30 min and observed under a fluorescence microscope. Cell nuclei were stained with Hoechst 33342, allowing for the simultaneous observation of the nuclei and ROS.

### 2.6. Cell Proliferation and Migration Assay

The effect of the GK-NVs on the proliferation of the BM-MSCs was assessed using the water-soluble tetrazolium-8 (WST-8, Biomax Inc., Seoul, Republic of Korea). The BM-MSCs were seeded in 96-well plates and cultured under serum-starved conditions using DMDM/F12 containing 0.5% fetal bovine serum (FBS) for 24 h. Following the UVB irradiation, the medium was replaced with DMEM/F12 containing GK-NVs and cultured for an additional 48 h. Cell viability was then evaluated by incubation with WST-8 solution for 2 h, and the absorbance was measured at 450 nm using a microplate reader. To evaluate the migratory potential, scratch closure and transwell migration assays were performed. In the scratch closure assay, the BM-MSCs were seeded in 24-well plates and subjected to serum starvation. After the treatment with GK-NVs in DMEM/F12, scratches were created using sterilized pipette tips following UVB irradiation 24 h post-GK-NV treatment. The closure of the gap was monitored under a microscope at 24 h intervals. For the transwell assay, the cells were seeded on an 8.0 μm porous membrane in transwell plates (Corning, Glendale, AZ, USA). The GK-NVs were applied to the lower chamber for 24 h. Cells that migrated to the bottom of the membrane were fixed with 4% paraformaldehyde (PFA) and stained with 0.5% crystal violet (CV, Sigma-Aldrich), and non-migrated cells on the top of the membrane were removed with a cotton swab. The stained cells were observed under a microscope and quantified by dissolving the CV in 50% acetic acid and measuring the absorbance at 560 nm using a microplate reader.

### 2.7. In Vitro Differentiation of BM-MSCs

To induce osteogenic differentiation, the BM-MSCs were plated at a density of 2 × 10^4^ cells/cm^2^ in 24-well plates. Following 24 h of incubation in growth medium, the medium was replaced with osteogenic medium composed of DMEM supplemented with 10% FBS, 10 mM glycerol phosphate, 50 μg/mL ascorbic acid, and 100 nM dexamethasone. This osteogenic medium was refreshed every two days without subculturing the cells. For the adipogenic differentiation, human BM-MSCs were seeded at a density of 4 × 10^4^ cells/cm^2^. After culturing in growth medium for 24 h, the medium was replaced with adipogenic medium consisting of DMEM supplemented with 10% FBS, 500 μM with 3-isobutyl-1-methylxanthine (IBMX), 0.1 μg/mL human insulin, and 1 μM dexamethasone. The adipogenic medium was refreshed every three days, also without subculturing. All of the supplements used for the BM-MSC differentiation were obtained from Sigma-Aldrich.

### 2.8. Alkaline Phosphatase (ALP) and Alizarin Red S (ARS) Staining

On day 9 of the osteoblast differentiation, the cells were fixed with 4% PFA and treated with the ALP staining kit II (Stemgent, Lexington, MA, USA) according to the manufacturer’s instructions to visualize the extracellular ALP activity. For the ARS staining, the cells on day 21 of osteogenesis were fixed, and the mineralized calcium deposits were stained with 2% ARS solution (Sigma-Aldrich). To quantify the deposits, 10% acetic acid was added to each stained well and incubated for 10 min. The dissolved stain was heated at 85 °C for 10 min, neutralized with 10% ammonium hydroxide, and the absorbance was measured at 405 nm using a microplate reader.

### 2.9. Von Kossa Staining and OsteoImage Mineralization Assay

Calcium deposition during osteogenesis was further assessed using Von Kossa staining and the OsteoImage mineralization assay. On day 21 of the osteoblast differentiation, the cells were fixed with 4% PFA, and the mineralized calcium deposits were stained using a Von Kossa staining kit (Abcam, Cambridge, UK) according to the manufacturer’s instructions. For the OsteoImage mineralization assay, the cells were fixed with 70% ethanol, followed by the addition of a fluorescent staining reagent (Lonza, Basel, Switzerland). The calcified matrices stained by the Von Kossa staining and the OsteoImage kit were subsequently examined under an optical microscope and a fluorescence microscope, respectively.

### 2.10. Oil Red O (ORO) Staining

Adipocyte differentiation of the BM-MSCs was examined using ORO staining. Differentiated cells were fixed with 4% PFA and subsequently stained with a 3:2 mixture of ORO solution (Sigma-Aldrich) and distilled water. For the quantitative analysis, 100% isopropyl alcohol was added to each stained well and incubated for 30 min. The absorbance of the dissolved staining solution was measured at 490 nm using a microplate reader.

### 2.11. Quantitative Real-Time Polymerase Chain Reaction (qPCR) Analysis

Total RNA was extracted from cells using the RiboEx™ LS (GeneAll Biotechnology Co., Ltd., Seoul, Republic of Korea) following the manufacturer’s instructions. The extracted RNA was reverse-transcribed into cDNA using the TOPScript™ RT DryMIX (Enzynomics, Daejeon, Republic of Korea). The qPCR was performed on a qTOWER3 system (Analytik Jena, Jena, Germany) utilizing TOPreal qPCR 2 × PreMIX (SYBR Green with low ROX; Enzynomics). Glyceraldehyde 3-phosphate dehydrogenase (GAPDH) served as the internal control. The primer sequences used in this study are summarized in Table 1. All of the experiments were conducted in triplicate, and the relative expression levels between the cell groups were calculated using the 2^−ΔΔCt^ method.

### 2.12. Statistical Analysis

All of the data are presented as the mean ± standard deviation (SD). Statistical analysis was performed by using a one-way analysis of variance (ANOVA) followed by Tukey’s post hoc test. Statistical significance between the experimental groups was defined as follows: * *p* < 0.05, ** *p* < 0.01, *** *p* < 0.005, and ns (not significant).

## 3. Results

### 3.1. Isolation and Characterization of GK-NVs

**Figure 1:**
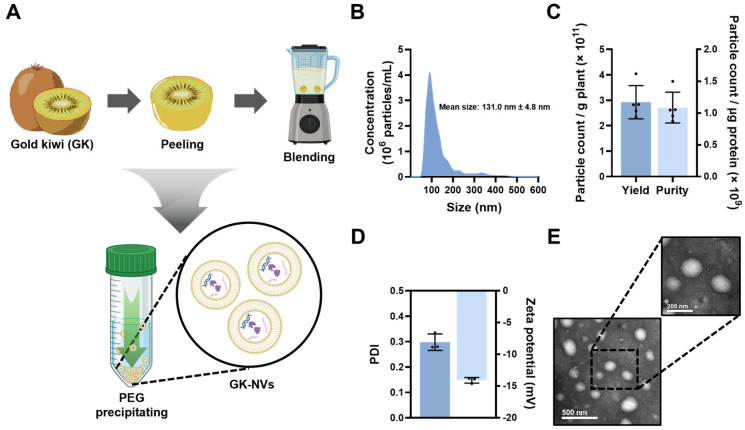
Isolation and characterization of the GK-NVs. (**A**) Schematic diagram illustrating the isolation process of the EVs from the gold kiwi. (**B**) Size distribution and mean size of the GK-NVs as determined by the NTA. (**C**) Quantification of the production yield and purity of the GK-NVs. The yield was calculated by dividing the total number of isolated GK-NV particles by the mass of gold kiwi used. The purity was determined by the number of particles per microgram of protein. (**D**) Zeta potential and PDI of the GK-NVs as measured by the DLS. (**E**) Morphology of the isolated GK-NVs as observed via TEM (created with BioRender.com).

In this study, the GK-NVs were isolated using a PEG-based precipitation technique, which has proven to be effective for processing large sample volumes (Figure 1A) [21]. When introduced to the extracted GK juice, PEG selectively reduces the solubility of the EVs, thereby facilitating their precipitation at lower gravitational forces. Consistent with the findings from our recent study, the GK juice was treated with 8% PEG and incubated for 16 h, followed by centrifugation at a standard speed rather than ultracentrifugation [21]. The NTA revealed that the isolated GK-NVs ranged in size from 50 to 150 nm, with an average diameter of approximately 131 nm (Figure 1B). The yield was 2.93 × 10^11^ EV particles per gram of gold kiwi, and the purity was 1.22 × 10^9^ EV particles per microgram of the total proteins (Figure 1C). The DLS analysis indicated that the zeta potential and the PDI of the GK-NVs were approximately −14 mV and 0.3, respectively (Figure 1D). Taken together with the TEM image, which revealed the spherical morphology of the 120 nm-sized GK-NVs (Figure 1E and Figure S1), these findings demonstrated that the GK-NVs are small, spherical particles with negatively charged cell membranes. Furthermore, we attempted to purify the EVs using ultracentrifugation following the PEG precipitation. However, this approach did not result in a significant increase in the purity (Figure S2).

### 3.2. Cytotoxicity and Cellular Uptake of GK-NVs

**Figure 2:**
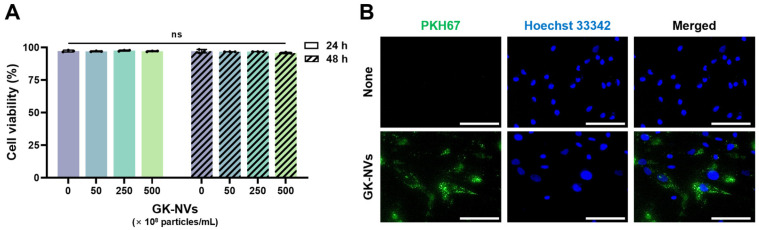
Cytotoxicity and cellular uptake of the GK-NVs. (**A**) Cytotoxicity assessment of the GK-NVs in the BM-MSCs using the trypan blue exclusion assay (ns: not significant; *n* = 3). (**B**) Cellular uptake of the GK-NVs by the BM-MSCs. BM-MSCs treated with PKH67-labeled GK-NVs were observed under a fluorescence microscope (scale bar = 100 µm).

Next, we treated human BM-MSCs with GK-NVs at a concentration up to 500 × 10^8^ particles/mL for 24 and 48 h to evaluate the cytotoxicity. The trypan blue exclusion assay showed no significant cytotoxicity across all of the tested concentrations and incubation durations (Figure 2A), which aligned with the results from our previous study on balloon flower root-derived EVs (BFR-EVs) [22]. Moreover, the BM-MSCs incubated with PKH67 dye-labeled GK-NVs for 12 h exhibited green fluorescence under a fluorescence microscope, indicating the successful intracellular uptake of the GK-NVs (Figure 2B). Collectively, these results suggest that GK-NVs can be delivered intracellularly into BM-MSCs without inducing significant cytotoxicity.

### 3.3. GK-NVs Attenuate UV-Induced Oxidative Stress and the Consequent Decline in Proliferative and Migratory Capacities of BM-MSCs

**Figure 3:**
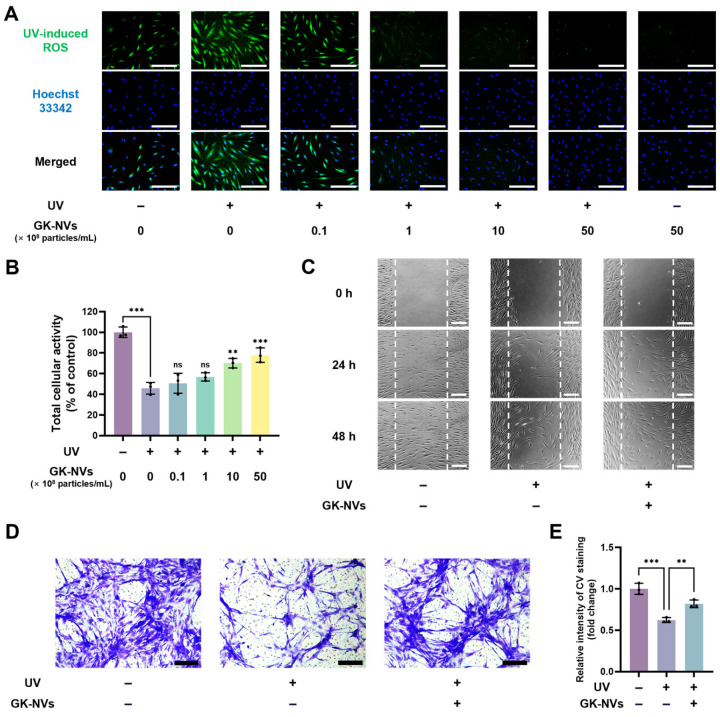
Effect of GK-NVs on the intracellular ROS level, proliferation, and migration of UVB-irradiated BM-MSCs. Following treatment with GK-NVs, the BM-MSCs were exposed to UVB irradiation (80 mJ/cm^2^) (scale bar = 200 µm). (**A**) Intracellular ROS levels assessed by the H2DCFDA assay. (**B**) Viable cell populations measured by the WST-8 assay (** *p* < 0.01 and *** *p* < 0.005; ns: not significant; *n* = 3). (**C**) Scratch closure assay demonstrating the migratory potential of BM-MSCs. The initial scratched area is indicated by the dashed line (scale bar = 200 µm). (**D**) Transwell migration assay showing the migration of the BM-MSCs treated with the GK-NVs, with migrated cells visualized by crystal violet staining (scale bar = 200 µm). (**E**) Quantification of the transwell migration assay. Statistical significance was determined by comparison to the UVB-irradiated, GK-NV-untreated control, unless otherwise noted (** *p* < 0.01 and *** *p* < 0.005; ns: not significant; *n* = 3).

In the previous study, it was observed that the treatment with BFR-EVs mitigated ROS generation induced by exposure to hydrogen peroxide and UV irradiation [22]. To investigate the effects of GK-NVs on cellular responses to oxidative stress, BM-MSCs were exposed to UVB irradiation at a wavelength of 315 nm using a 6W lamp positioned 12 cm above the cells for 24 s. Intracellular ROS were visualized by H2DCFDA staining. The results demonstrated the treatment with the GK-NVs significantly attenuated the intracellular ROS levels in a dose-dependent manner (Figure 3A). Notably, doses exceeding 10 × 10^8^ particles/mL resulted in ROS levels lower than those observed in the control group, which was not exposed to UVB irradiation. Moreover, at a concentration of 50 × 10^8^ particles/mL, the GK-NV treatment consistently maintained intracellular ROS levels across both the UVB-irradiated and non-irradiated conditions. Although the precise mechanism by which GK-NVs reduce ROS levels is not known, these findings suggest that the GK-NV treatment effectively reduces the intracellular ROS level induced by UV irradiation.

UV irradiation not only directly damages DNA but also induces intracellular oxidative stress, leading to indirect DNA damage, both of which contribute to genomic instability and play a critical role in aging. This process results in a decline in various cellular functions, including proliferation and migration [26,27]. Subsequently, we explored whether GK-NVs could mitigate the effects of UV irradiation on the proliferation and migration of BM-MSCs. Preliminary studies indicated that GK-NVs stimulate the proliferation of BM-MSCs under normal conditions (Figure S3A). Accordingly, the BM-MSCs were pre-treated with GK-NVs, followed by exposure to UVB. The WST-8 assay revealed a significant reduction in the total viable cells to approximately 50% of the levels observed in the non-irradiated control levels; however, the GK-NV treatment restored the cell viability in a dose-dependent manner (Figure 3B). In the scratch closure assay, a time-course analysis revealed that the treatment with the GK-NVs at a concentration of 50 × 10^8^ particles/mL significantly enhanced the rate of cell migration in both the UVB-irradiated (Figure 3C) and non-irradiated BM-MSCs (Figure S3B). Furthermore, the impact of GK-NVs on cell migration was investigated by a transwell migration assay. The CV staining of the cells that migrated across the membrane indicated that cellular motility, which was reduced by the UVB irradiation, was partially restored by the GK-NV treatment (Figure 3D). Quantitative analysis of the CV staining further demonstrated that UVB irradiation decreased BM-MSC migration by approximately 40% relative to unirradiated controls, but treatment with GK-NVs recovered these levels to about 90% of those in control conditions (Figure 3E). These findings suggest that UVB-induced photoaging in BM-MSCs, which impairs cellular proliferation and migration, may potentially be ameliorated by treatment with GK-NVs.

### 3.4. GK-NVs Enhance the Osteogenic Differentiation of BM-MSCs Subjected to UV-Induced Photoaging

**Figure 4:**
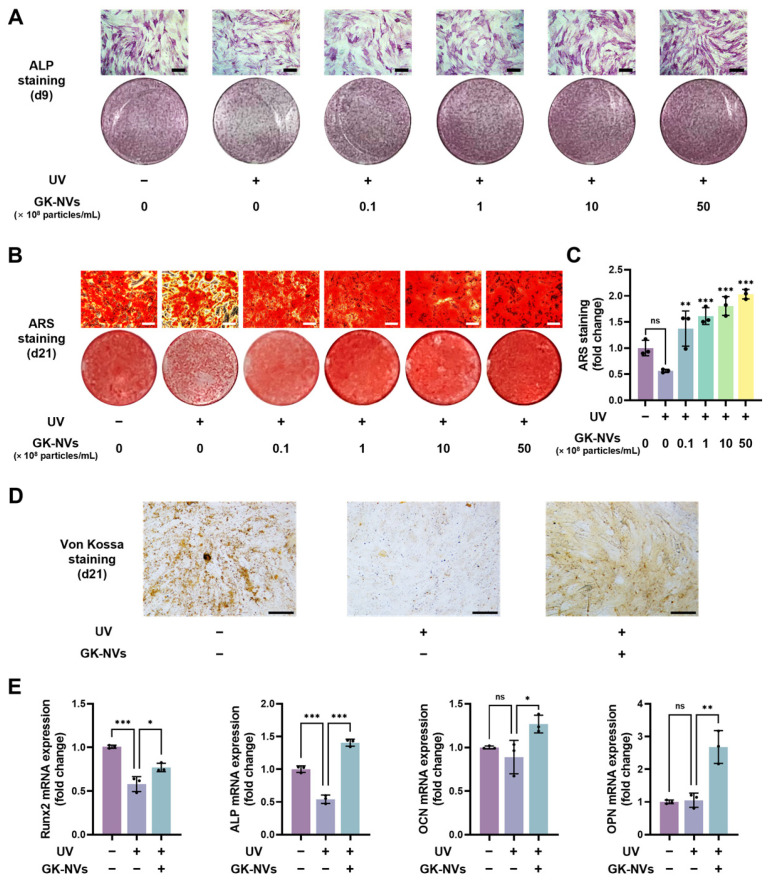
Effects of GK-NVs on the osteoblast differentiation BM-MSCs exposed to UVB irradiation. UVB-irradiated BM-MSCs were treated with GK-NVs twice during the first week of osteogenesis. (**A**) Representative images of the ALP staining on day 9 of the osteogenic culture (scale bar = 200 µm). (**B**) Representative image of the ARS staining demonstrating calcium deposition on day 21 of the osteogenic culture (scale bar = 200 µm). (**C**) Quantification of the ARS staining (** *p* < 0.01 and *** *p* < 0.005; ns: not significant; *n* = 3). (**D**) Representative image of the Von Kossa staining on day 21 of the osteogenic culture (scale bar = 200 µm). (**E**) The mRNA expression levels of osteoblast-specific genes, including ALP, Runx2, OPN, and OCN. A qPCR analysis was conducted using the total RNA extracted from the BM-MSCs on day 14 of the osteogenic culture. Statistical significance was determined by comparison to the UVB-irradiated, GK-NV-untreated control, unless otherwise noted (* *p* < 0.05, ** *p* < 0.01, and *** *p* < 0.005; ns: not significant; *n* = 3).

Based on the observation that the GK-NVs restored the impaired cellular function of UVB-induced photoaged BM-MSCs, we then evaluated their effect on the osteogenic differentiation of the BM-MSCs. First, we determined the expression of the early osteoblast marker, alkaline phosphatase (ALP), at day 9 of the differentiation of the photoaged BM-MSCs. The ALP expressions, diminished by the UVB irradiation, were restored by the GK-NV treatment in a dose-dependent manner up to 50 × 10^8^ particles/mL (Figure 4A). The restoration of osteogenic potential by the GK-NV treatment was further evidenced by the quantification of calcium deposits, a hallmark of osteoblast differentiation and bone nodule formation. Osteoblasts contribute to bone formation through mineralization, which involves the deposition of extracellular calcium deposits [28]. Similar to the results observed with the ALP staining, the ARS staining indicated that calcium deposition at the late stage of differentiation (day 21) was reduced by the UVB irradiation but was restored by the GK-NV treatment (Figure 4B). Quantitative analysis for the ARS staining exhibited that the calcium matrices stained with ARS were elevated in the UVB-irradiated BM-MSCs in a dose-dependent manner upon treatment with GK-NVs (Figure 4C). Higher rates of mineralization were observed with GK-NV concentrations of 1 × 10^8^ particles/mL and above compared to cells without UVB exposure. The augmented mineralization with the GK-NV treatment in the UVB-exposed BM-MSCs was also confirmed using Von Kossa (Figure 4D) and OsteoImage fluorescence staining (Figure S4). To evaluate whether the GK-NVs influence osteogenic differentiation in normal BM-MSCs, we treated non-UVB-irradiated BM-MSCs with GK-NVs and induced osteogenic differentiation. The ARS staining revealed no significant difference in the calcium deposition between the GK-NV-treated and untreated control BM-MSCs (Figure S5). These findings suggest that GK-NVs do not significantly impact osteogenic differentiation in normal BM-MSCs, indicating that their effects are more pronounced under conditions of UVB-induced photoaging.

In addition, we examined the effects of GK-NVs on the osteogenic differentiation of UVB-irradiated BM-MSCs at the transcriptional level (Figure 4E). We assessed the mRNA levels of osteoblast-specific genes, including runt-related transcriptional factor 2 (Runx2), ALP, osteocalcin (OCN), and osteopontin (OPN), via qPCR analysis. These were compared with a control group that was not treated with GK-NVs. It was shown that UVB irradiation reduced the expression of Runx2 and that ALPs were decreased by UVB exposure; however, this reduction was significantly reversed upon treatment with GK-NVs. Notably, the ALP expression in the GK-NV-treated group post-UVB exposure was higher than in the UVB-unexposed controls. Similarly, the expressions of OCN and OPN were significantly decreased by UVB exposure but were substantially increased upon the GK-NV treatment. These results paralleled the findings from the ARS staining, indicating that the GK-NV treatment restored the osteogenic potential diminished by the UVB exposure. Collectively, these findings suggest that GK-NVs promote the osteogenesis of BM-MSCs subjected to UV-induced photoaging by influencing the osteogenic pathways at the transcriptional level.

### 3.5. GK-NVs Inhibited the Adipocyte Differentiation of BM-MSCs Subjected to UV-Induced Photoaging

**Figure 5:**
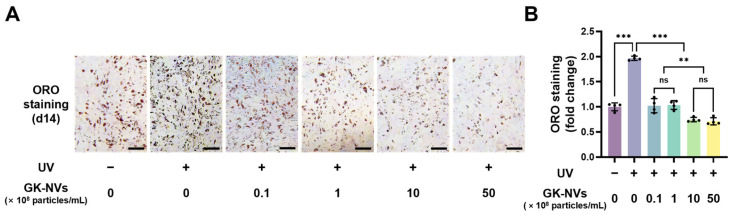
Effects of GK-NVs on the adipocyte differentiation BM-MSCs exposed to UVB irradiation. UVB-irradiated BM-MSCs were treated with GK-NVs twice during the first week of adipogenesis. (**A**) Representative image of the ORO staining demonstrating the formation of lipid droplets on day 14 of the adipogenic culture (scale bar = 200 µm). (**B**) Quantification of the ORO staining. Statistical significance was determined with a comparison to the UVB-irradiated, GK-NV-untreated control, unless otherwise noted (** *p* < 0.01 and *** *p* < 0.005; ns: not significant; *n* = 3).

Next, we examined whether treatment with GK-NVs suppresses the adipogenesis of BM-MSCs, which was stimulated by UVB irradiation, while simultaneously enhancing osteogenesis. Adipogenesis was evaluated using ORO staining, a straightforward method for quantifying lipid droplets in adipocytes. On day 14 of adipocyte differentiation, representative ORO staining images showed that UVB irradiation significantly increased the size and number of lipid droplets, whereas the treatment with the GK-NVs effectively inhibited UV-induced adipogenesis in the BM-MSCs (Figure 5A). Furthermore, the quantitative analysis for the ORO staining revealed that the lipid droplets decreased significantly in a dose-dependent manner following treatment with the GK-NVs (Figure 5B). Treatments with a concentration of 10 × 10^8^ particles/mL or higher of GK-NVs resulted in reduced adipogenesis compared to the UV-untreated control, likely due to the reduced intracellular ROS levels in the GK-NV-treated BM-MSCs relative to the control cells cultured without UV irradiation. To further evaluate the effects of the GK-NVs on adipogenesis, we treated the BM-MSCs not subjected to UVB irradiation with GK-NVs and induced adipogenic differentiation. The ORO staining revealed a statistically significant, yet relatively modest, reduction in lipid accumulation compared to the untreated controls (Figure S6). Consistent with the observation in the osteogenesis, these results suggest that GK-NVs have a limited inhibitory effect on adipogenesis in normal BM-MSCs, indicating that their effects are more pronounced under conditions of UVB-induced photoaging. When considered in conjunction with the osteogenesis results, these findings suggest that GK-NVs inhibit photoaging-induced skewness in the lineage commitment towards adipocytes and restore the osteogenic potential of the BM-MSCs.

## 4. Discussion

Accumulated oxidative stress can damage various intracellular biomolecules, particularly DNA, leading to cellular aging [29]. Numerous studies have demonstrated that an MSC-like population within bone marrow cavities is capable of giving rise to both osteoblast and adipocyte lineages, and that aging reprograms these populations, favoring adipocyte differentiation while inhibiting osteogenesis [30,31]. This shift in lineage commitments is implicated in compromised bone regeneration and an increased fracture risk associated with age-related osteoporosis [6]. Given the impact of ROS generation and phenotypic alterations induced by photoaging, we hypothesized that GK-NVs could restore the osteogenic potential of BM-MSCs impaired by UV irradiation.

Recent studies have reported that pEVs can modulate various cellular functions, including proliferation, apoptosis, and differentiation. These activities of pEVs are attributed to the unique bioactive compounds and nucleic acids (e.g., small RNAs) they contain, as determined by chemical properties. Research has shown that the anti-inflammatory effects of ginger-derived EVs in a colitis model are due to the key bioactive compounds of ginger, such as 6-gingerol and 6-shogaol, which are also present in the EVs [32]. In addition, studies of strawberry and *Citrus limon* L. EVs have reported the presence of antioxidant compounds, such as vitamin C, which alleviate the oxidative stress in MSCs [19,33]. Furthermore, strawberry-derived EVs were found to contain both vitamin C and small RNAs with active biological functions. Compared to other fruits, gold kiwi has a significantly higher Oxygen Radical Absorbance Capacity (ORAC) value, which is due to its particularly high vitamin C content from the same amount of fruits [34,35]. These findings suggest that GK-NVs may also encapsulate high levels of antioxidant compounds and functional small RNAs inherent to gold kiwi. Such constituents could potentially contribute to the capacity of GK-NVs to modulate cellular functions, including restoring the osteogenic differentiation potential in BM-MSCs. Unlike the high-dose supplementation of antioxidants such as vitamins C and E, GK-NVs offer enhanced stability and efficient cellular uptake due to their lipid bilayer structure. These properties not only protect the encapsulated antioxidants from degradation but also enable the GK-NVs to modulate cellular antioxidant pathways more effectively [12,36,37]. In this study, we isolated the GK-NVs using a simple precipitation method that we previously optimized. The PEG-based precipitation technique offers a more time- and cost-efficient alternative to the widely used ultracentrifugation method by effectively reducing the initial sample volume [21]. The biological efficacy of the isolated GK-NVs was then evaluated in an aging model of BM-MSCs. We systematically investigated the impact of these GK-NVs on various cellular functions of aging BM-MSCs, including the ROS levels, cell migration, proliferation, and differentiation, to assess their potential therapeutic efficacy.

We induced aging in the BM-MSCs through UVB irradiation, a well-established method for promoting photoaging. Numerous studies have shown that UVB irradiation leads to the physiological deterioration in skin cells through photoaging, directly damaging intracellular molecules such as DNA and proteins, while also indirectly causing damage through oxidative stress [38,39]. Meanwhile, some recent studies on EVs have shown their potential to mitigate UV-induced photoaging by scavenging ROS and normalizing the expression levels of senescence markers such as p53, p21, and SA-β-Gal. For example, EVs derived from *Phellinus linteus* (PL) were found to effectively scavenge significant amounts of ROS generated in UV-irradiated skin cells, demonstrating their anti-aging properties, as evidenced by the reduced SA-β-gal levels [40]. Consistent with these findings, the GK-NVs demonstrated antioxidative activity by effectively scavenging ROS generated in the BM-MSCs after UVB irradiation. Our results suggest that the increase in ROS levels induced by the UVB exposure in the BM-MSCs triggered alterations in the expression of aging-related markers, which the GK-NVs can restore by mitigating the oxidative stress. In addition, UVB exposure resulted in a significant decline in cellular proliferation and migration abilities, along with an altered differentiation tendency, indicating that UVB irradiation induces physiological changes in BM-MSCs similar to those observed in natural senescence, thereby reflecting the effect of photoaging in BM-MSCs.

The isolated GK-NVs readily penetrated the BM-MSCs without significant cytotoxicity. Although both the cell membrane and EVs are negatively charged, leading to electrostatic repulsion, this does not impede the cellular uptake of EVs, as interactions mediated by surface molecules such as integrins and tetraspanins effectively overcome this barrier [36,37]. Upon UVB irradiation, the GK-NVs reduced the intracellular ROS levels, thereby protecting the BM-MSCs from oxidative stress and restoring their impaired proliferation and migration capacities. Elevated ROS levels are known to skew the lineage commitment towards adipogenesis at the expense of osteogenesis through pathways such as MAPK and PPARγ [41,42]. Moreover, several studies have demonstrated that inhibiting ROS through antioxidant mechanisms involving pathways such as Nrf2 is crucial for alleviating bone loss in both in vitro and in vivo models [43]. The GK-NV treatment also corrected the skewed differentiation of the BM-MSCs caused by the UV irradiation, enhancing osteoblast differentiation, as indicated by the increased expression in the osteoblast-specific genes and calcium deposition. At the same time, the reduced lipid droplet formation indicated decreased adipocyte differentiation. At higher concentrations, the treatment with the GK-NVs resulted in higher osteoblast and lower adipocyte differentiation compared to the UV-untreated control. These results are suggested to result from the ability of GK-NVs to mitigate not only UVB-induced oxidative stress but also the oxidative stress that naturally occurs in cultured BM-MSCs. This hypothesis is further supported by the H2DCFDA staining results, which demonstrated that the GK-NV treatment post-UVB exposure resulted in significantly lower intracellular ROS levels compared to the UVB-untreated cells. Our findings suggest that GK-NVs reduce oxidative stress in BM-MSCs, which subsequently alleviates aging-related impairments in cellular functions associated with bone regeneration.

The primary limitation of this study is the lack of a mechanistic insight into how GK-NVs reduce oxidative stress and affect the lineage commitment in BM-MSCs. However, previous studies provide valuable insights into the two primary mechanisms by which pEVs mitigate oxidative stress through their inherent bioactive compounds and small RNAs. These mechanisms include the following: (1) the direct scavenging of intracellular ROS by natural antioxidants (e.g., vitamin C) and small RNAs present in the pEVs, as shown in those derived from strawberry, and (2) the modulation of cellular signaling pathways that enhance the expression of antioxidant enzymes and related genes, including catalase, SOD, and Nrf2 [17]. For example, research on carrot-derived EVs has demonstrated their ability to restore the Nrf2 pathway and increase the expression of antioxidant proteins, such as HO-1 and NQO-1, in H9C2 cardiomyocytes and SH-SY5Y neuroblastoma cells, thereby regulating intracellular ROS levels [44]. While specific biomolecules among these constituents may play a critical role in the antioxidative properties, it is also possible that the observed beneficial effects of GK-NVs result from synergistic interactions among multiple unique constituents. Future effort should investigate whether GK-NVs directly scavenge intracellular ROS or indirectly protect from oxidative stress by modulating intracellular antioxidant machinery. Identifying GK-NV components at the molecular level will further clarify the mechanism of action.

Moreover, batch-to-batch variability in the production of GK-NVs may pose challenges for their scalability and industrial application. This variability could impact the product consistency and therapeutic efficacy, underscoring the necessity for robust quality control measures. Future studies should focus on standardizing manufacturing processes to minimize such variability, ensuring that GK-NVs can be reliably produced at scale for clinical use. Addressing these issues is essential for translating GK-NVs into viable therapeutic agents for oxidative stress-related conditions.

## 5. Conclusions

In summary, our findings indicate that GK-NVs inhibit the UV-induced elevation of intracellular ROS levels, enhancing the osteogenic capacity while reducing the adipocyte differentiation in BM-MSCs. These results suggest the potential of GK-NVs to restore the differentiation potential of BM-MSCs impaired by photoaging, which is of great importance from the perspective of agricultural and food engineering. In addition, considering the global popularity and substantial market presence of gold kiwi, our research highlights the functional potential of GK-NVs for developing novel nutraceuticals or functional foods targeting age-related disorders such as osteoporosis. Notably, recent studies have demonstrated that orally administered pEVs can effectively reach various organs, including bone tissue [45]. Therefore, further in vivo studies on the distribution and efficacy of GK-NVs in bone tissue will be essential to confirm their therapeutic potential.

## Figures and Tables

**Table 1:** List of specific primers used for the qPCR.

Gene (Accession #)	Primer	Sequence (5′-3′)
GAPDH (NM_001357943.2)	Sense	GTC AGT GGT GGA CCT GAC CT
	Antisense	TGC TGT AGC CAA ATT CGT TG
Runx2 (NM_001369405.1)	Sense	GTC TTA CCC CTC CTA CCT GA
	Antisense	TGC CTG GCT CTT CTT ACT GA
ALP (XM_054335748.1)	Sense	ACG TGG CTA AGA ATG TCA TC
	Antisense	CTG GTA GGC GAT GTC CTT A
OCN (NM_199173.6)	Sense	CAA AGG TGC AGC CTT TGT GTC
	Antisense	TCA CAG TCC GGA TTG AGC TCA
OPN (NM_000582.3)	Sense	GTT TCG CAG ACC TGA CAT CC
	Antisense	CAT TCA ACT CCT CGC TTT CC

## Data Availability

The original contributions presented in the study are included in the article and Supplementary Materials; further inquiries can be directed to the corresponding author.

## Supplementary Materials

The following supporting information can be downloaded at: https://www.mdpi.com/article/10.3390/antiox13121474/s1, Figure S1: Morphology of a single GK-NV particle observed with a high-resolution image via TEM; Figure S2: Comparison of the purity quantification across GK-NV isolation techniques; Figure S3: Promotion of proliferation and migration by GK-NVs in BM-MSCs; Figure S4: Representative images of the OsteoImage mineralization analysis showing the formation of calcium deposits; Figure S5: Representative image of the ARS staining demonstrating calcium deposition on day 21 of the osteogenic culture; Figure S6: Effects of the GK-NVs on the adipocyte differentiation BM-MSCs. The BM-MSCs were treated with GK-NVs twice during the first week of adipogenesis.
