# Supplementary material for: Gold Kiwi-Derived Nanovesicles Mitigate Ultraviolet-Induced Photoaging and Enhance Osteogenic Differentiation in Bone Marrow Mesenchymal Stem Cells
Source: Antioxidants (Basel). 2024 Nov 29;13(12):1474. doi: 10.3390/antiox13121474 (PMC11673108; doi:10.3390/antiox13121474)
Supplement: Supplementary file 1 [file antioxidants-13-01474-s001.zip › antioxidants-3310079-supplementary.pdf]

Supplementary Materials

Gold kiwi-derived nanovesicles mitigate ultraviolet-induced photoaging and enhance osteogenic differentiation in bone marrow mesenchymal stem cells

Doyeon Kim [1,†], Chanho Lee [1,†],Manho Kim [1] and Ju Hyun Park [1,2,*]

[1] Department of Biomedical Science, Kangwon National University, Chuncheon-si, Gangwon-do 24341, Republic of Korea; holly990302@gmail.com (D.K.); cksgh1006@gmail.com (C.L.); manhokim@kangwon.ac.kr (M.K.)

[2] Institute of Molecular Science and Fusion Technology, Kangwon National University, Chuncheon 24341, Republic of Korea

* Correspondence: juhyunpark@kangwon.ac.kr; Tel.: +82-33-250-6566; Fax: +82-33-259-5645

† These authors contributed equally to this work.

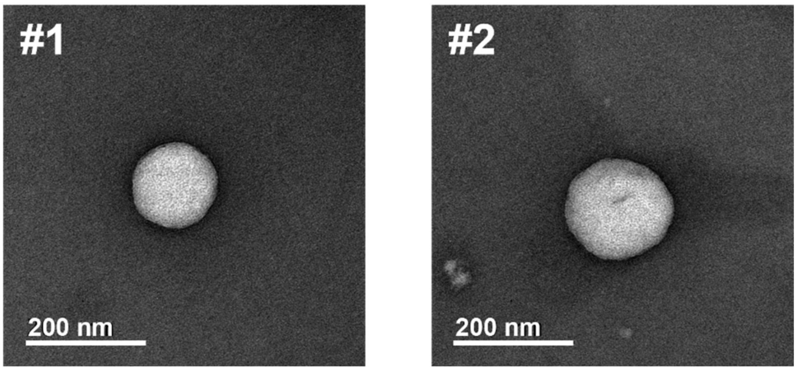

Figure S1. Morphology of a single GK-NVs particle observed with a high-resolution image via TEM.

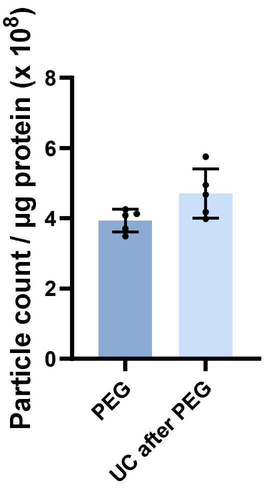

Figure S2. Comparison of purity quantification across GK-NVs isolation techniques. The analysis revealed no significant difference in purity between samples treated with PEG alone and those subjected to PEG treatment followed by ultracentrifugation (UC) (* $p < 0.05$, $n = 3$).

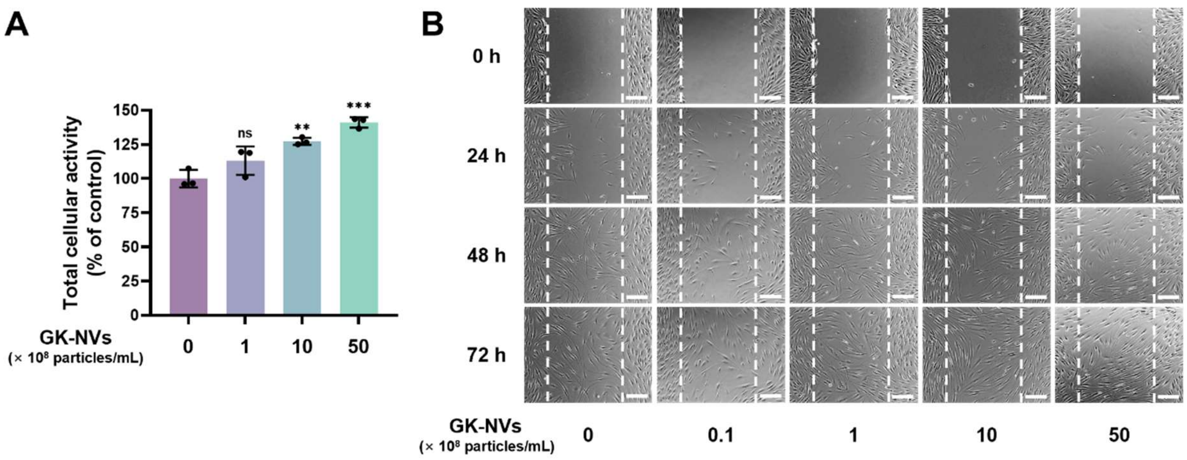

Figure S3. Promotion of proliferation and migration by GK-NVs in BM-MSCs. (**A**) BM-MSCs were serum-starved and then treated with GK-NVs for 48 h in serum-free DMEM/F12 medium. Viable populations of BM-MSCs were determined using the WST-8 assay. Statistical significance was determined by comparison to GK-NV-untreated control, unless otherwise noted (** $p < 0.01$, *** $p < 0.005$, ns: not significant, $n = 3$). (**B**) Effect of GK-NVs on migration of BM-MSCs was evaluated using the scratch closure assay. The initial scratched area is indicated by the dashed line (scale bar = 200 μm).

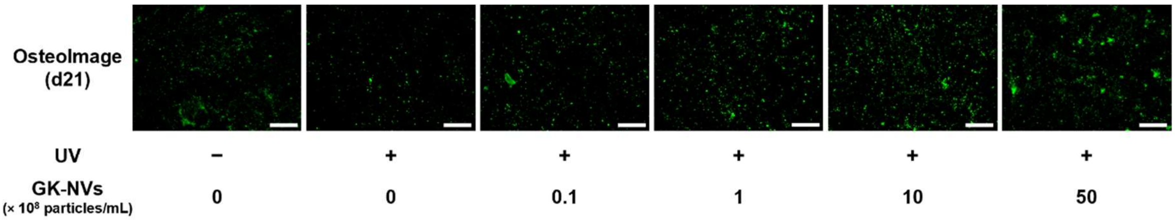

Figure S4. Representative images of OsteoImage mineralization analysis showing the formation of calcium deposits. UVB-irradiated BM-MSCs were treated with GK-NVs twice during the first week of osteogenesis. On day 21 of osteogenesis, the cells were stained and observed under a fluorescence microscope (scale bar = 200 μm).

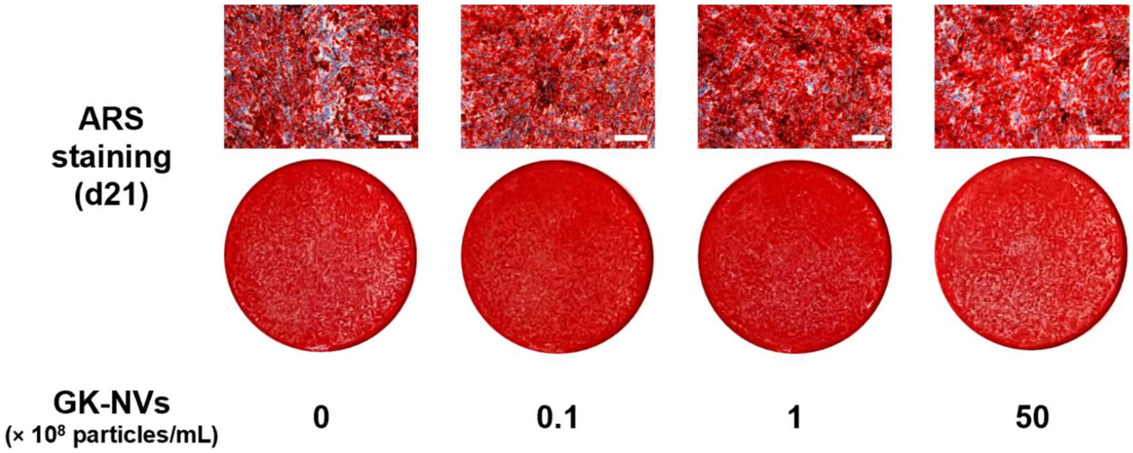

Figure S5. Representative image of ARS staining demonstrating calcium deposition on day 21 of osteogenic culture. BM-MSCs were treated with GK-NVs twice during the first week of osteogenesis. On day 21 of osteogenesis, the cells were stained and observed under a fluorescence microscope (scale bar = 200 μm).

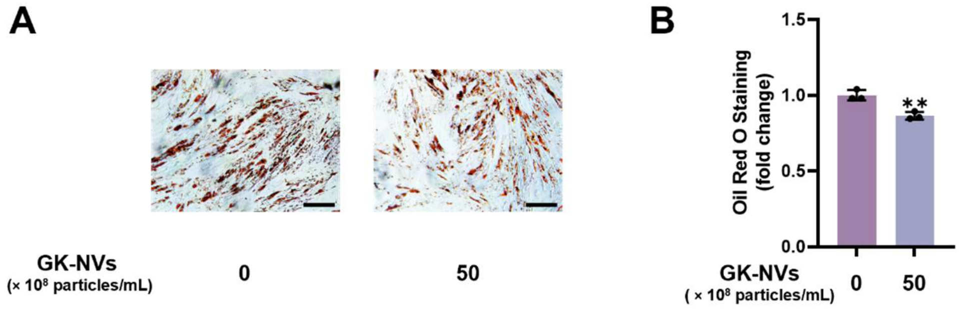

Figure S6. Effects of GK-NVs on the adipocyte differentiation BM-MSCs. BM-MSCs were treated with GK-NVs twice during the first week of adipogenesis. (A) Representative image of ORO staining demonstrating the formation of lipid droplets on day 14 of adipogenic culture (scale bar = 200 μm). (B) Quantification of ORO staining. Statistical significance was determined by comparison to GK-NV-untreated control, unless otherwise noted (** $p < 0.01$, $n = 3$).
